# Dr. George A. Campbell

**Published:** 1915-04

**Authors:** 


**Dr. George A. Campbell,** formerly of Buffalo, died in Wheeling, West Virginia, last month.

